# *cis*-6-Bromo-4-(1-methyl-1*H*-indol-3-yl)-10,10a-dihydro-1*H*,4*H*-2,9-dioxa-3-aza­benz[*f*]azulene

**DOI:** 10.1107/S1600536811003084

**Published:** 2011-01-29

**Authors:** P. Narayanan, K. Sethusankar, K. Ramachandiran, P. T. Perumal

**Affiliations:** a Department of Physics, RKM Vivekananda College (Autonomous), Chennai 600 004, India; b Organic Chemistry Division, Central Leather Research Institute, Adyar, Chennai 600 020, India

## Abstract

In the title compound, C_20_H_17_BrN_2_O_2_, the seven-membered oxepine ring adopts a chair conformation. The indole moiety is essentially planar with a maximum deviation of 0.031 (3)Å. The indole ring system forms a dihedral angle of 21.87 (8)° with the mean plane of the 10-membered heterobicycle. The crystal packing is stabilized by inter­molecular C—H⋯O and C—H⋯π inter­actions.

## Related literature

For the chemistry of 4,5-dihydro­isoxazole, see: Caramella & Grunanger (1984 ▶). For the uses of isoxazoline derivatives, see: Ichiba & Scheuer (1993 ▶). For intra­molecular nitrile oxide cyclo­addition (INOC) reactions, see: Scott *et al.* (2006 ▶); Mukaiyama & Hoshino (1960 ▶). For a related structure, see: Trigunait *et al.* (2010 ▶). For puckering and asymmetry parameters, see: Cremer & Pople (1975 ▶); Nardelli (1983 ▶). For bond-length distortions, see: Allen (1981 ▶).
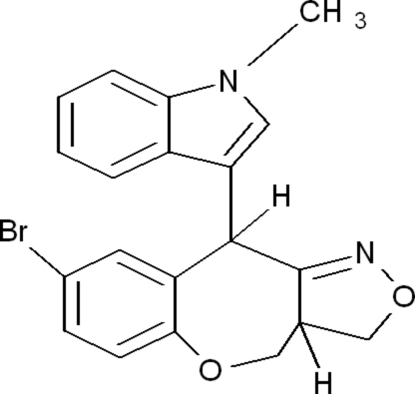


## Experimental

### 

#### Crystal data

C_20_H_17_BrN_2_O_2_
*M* *_r_* = 397.26
Monoclinic, 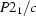

*a* = 12.5772 (4) Å
*b* = 14.7746 (5) Å
*c* = 9.2168 (3) Å
β = 97.193 (2)°
*V* = 1699.22 (10) Å^3^
*Z* = 4
Mo *K*α radiation
μ = 2.44 mm^−1^
*T* = 295 K
0.30 × 0.20 × 0.20 mm

#### Data collection

Bruker Kappa APEXII CCD diffractometer
21255 measured reflections
4461 independent reflections
2831 reflections with *I* > 2σ(*I*)
*R*_int_ = 0.041

#### Refinement

*R*[*F* ^2^ > 2σ(*F* ^2^)] = 0.039
*wR*(*F* ^2^) = 0.099
*S* = 1.00
4461 reflections
227 parameters
H-atom parameters constrained
Δρ_max_ = 0.45 e Å^−3^
Δρ_min_ = −0.38 e Å^−3^

### 

Data collection: *APEX2* (Bruker, 2004 ▶); cell refinement: *SAINT* (Bruker, 2004 ▶); data reduction: *SAINT*; program(s) used to solve structure: *SHELXS97* (Sheldrick, 2008 ▶); program(s) used to refine structure: *SHELXL97* (Sheldrick, 2008 ▶); molecular graphics: *ORTEP-3* (Farrugia, 1997 ▶); software used to prepare material for publication: *SHELXL97* and *PLATON* (Spek, 2009 ▶).

## Supplementary Material

Crystal structure: contains datablocks global, I. DOI: 10.1107/S1600536811003084/rk2253sup1.cif

Structure factors: contains datablocks I. DOI: 10.1107/S1600536811003084/rk2253Isup2.hkl

Additional supplementary materials:  crystallographic information; 3D view; checkCIF report

## Figures and Tables

**Table 1:** Hydrogen-bond geometry (Å, °) *Cg*1and *Cg*2 are the centroids of the C1–C6 and C15–C20 rings, respectively.

*D*—H⋯*A*	*D*—H	H⋯*A*	*D*⋯*A*	*D*—H⋯*A*
C17—H17⋯O1^i^	0.93	2.53	3.329 (3)	144
C3—H3⋯*Cg*2^ii^	0.93	2.79	3.653 (3)	155
C7—H7*A*⋯*Cg*1^iii^	0.96	2.92	3.427 (3)	114

Symmetry codes: (i) 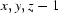; (ii) 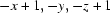; (iii) 
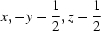
.

**Fig. 1.**
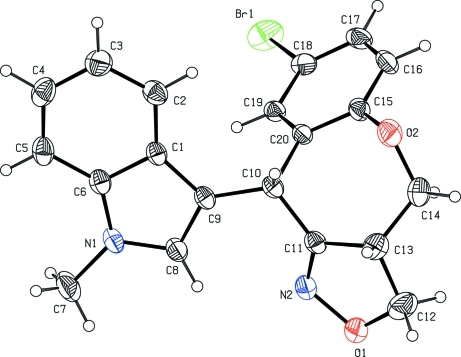
The molecular structure of the title compound with the atom numbering scheme. Displacement ellipsoids are drawn at 30% probability level. H atoms are present as small spheres of arbitary radius.

**Fig. 2.**
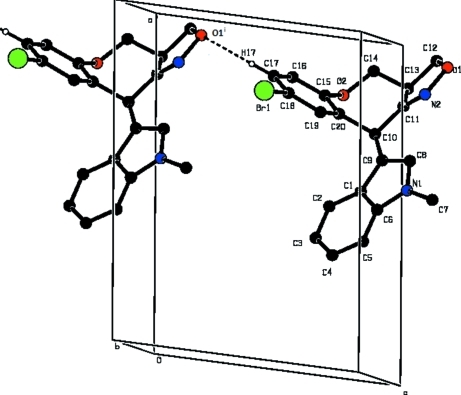
The packing arrangement of the title compound. Dashed lines indicates the C–H···O interactions. Symmetry code: (i) x, y, z-1.

## supplementary crystallographic information

### Comment

1,3-Dipolar cycloaddition of nitrile oxide is regarded as a useful synthetic method in organic synthesis. The cycloaddition occurs in a stereospecific way to provide 4,5-dihydroisoxazoles (Caramella & Grunanger, 1984). The isoxazoline derivatives are known to exibit interesting biological activities in the agricultural field and possess medicinal properties including antiviral and anti-HIV activities (Ichiba & Scheuer, 1993). Intramolecular nitrile oxide cycloaddition (INOC) reaction offers a powerful strategy to construct carbo- or heterocyclic compounds and many reports on the stereoselective INOC reaction have been published in recent years (Scott *et al.*, 2006). The nitro group is converted into nitrile oxide by Mukaiyama reaction (Mukaiyama & Hoshino, 1960). They successfully prepared five- and six-membered carbo-, oxa-, and thiocyclic compounds and one-pot conversion of nitroalkenes to bicyclic isoxazoles was utilized in some protocols.

The molecular structure of the title compound C_20_H_17_BrN_2_O_2_, is shown at Fig. 1. In the compound, the indole bicycle is essentially planar. The maximum deviation of the atom C7 of the methyl group from the indole bicycle is 0.031 (3)Å. The indole moiety (N1/C1/C2/C3/C4/C5/C6/C8/C9) forms the dihedral angles with the main plane of heterobicycle (O1/O2/N2/C10/C11/C12/C13/C14/C15/C20) and phenyl ring (C15-C20) 21.85 (8)° and 71.10 (10)°, respectively.

In the benzene ring of indole moiety, the *endo*-cyclic angles at C5 and C2 are contracted to 117.8 (2)° and 118.9 (2)°, respectively, while those at C6, C4 and C3 are expanded to 121.9 (2)°, 121.6 (3)° and 121.2 (3)°, respectively. This would appear to be a real effect caused by the fusion of the smaller pyrrole ring to the six-membered benzene ring, and the strain is taken up by the angular distortion rather than by bond-length distortions (Allen, 1981).

The dihedral angle between the phenyl ring (C15-C20) and main plane of heterobicycle (O1/O2/N2/C10/C11/C12/C13/C14/C15/C20) is 50.91 (9)°. The dihedral angle between the phenyl ring (C15-C20) and five-membered oxazole ring (C11/N2/O1/C12/C13) is 73.83 (11)°. The maximum deviation of the atom Br1 from the phenyl ring (C15-C20) is 0.0635 (4)Å. The angles around atom C10 [C9–C10–C20 = 113.84 (18)°, C11–C10–C9 = 116.65 (18)° and C11–C10–C20 = 107.06 (17)°] deviates significantly from ideal tetrahedral values which may be as a result of steric interactions between isoxazole, bromophenol and indole moieties (Trigunait *et al.*, 2010).

The oxepine ring (C10/C11/C13/C14/O2/C15/C20) adopts a *chair* conformation with puckering parameters (Cremer & Pople, 1975 and Nardelli, 1983) of q_2_ = 0.547 (2)Å, φ_2_ = 158.6 (3)°, q_3_ = 0.602 (2)Å, φ_3_ = 232.4 (2)°. Also, the maximum deviation of atoms C10 and O2 of the oxepine ring are -0.0458 (2)Å and -0.469 (2)Å, respectively. The main plane of oxepine ring forms a dihedral angle with the indole ring system 35.42 (9)°. The position of atom O2 which lies between bromobenzene and isoxazone rings is defined by torsion angles O2–C15–C16–C17 = -178.4 (2)° and C12–C13–C14–O2 = -166.4 (2)°.

The crystal packing is stabilized by intermolecular C–H···O- and C–H···π-interactions, where *Cg*1 is the center of gravity of C1/C2/C3/C4/C5/C6 ring and *Cg*2 is the center of gravity of C15/C16/C17/C18/C19/C20 ring; The symmetry codes: (i) *x*, *y*, *z*-1; (ii) -*x*+1, -*y*, -*z*+1; (iii) *x*, -*y*-1/2, *z*-1/2. The packing view of the title compound is shown at Fig. 2.

### Experimental

3-[1-(2-Allyloxy-5-bromo-phenyl)-2-nitro-ethyl]-2-methyl-1 *H*-indole (1.0 mmol) and *N*,*N*-dimethyl-4-aminopyridine (0.2 mmol) were dissolved in toluene (5 ml). Di-*tert*-butyl dicarbonate (2.5 mmol) in toluene (5 ml) was added in portions over a period of 0.5 h at 363 K to the nitroalkane solution and the reaction was allowed to proceed for a further 2 h. The mixture was evaporated and the product was purified by column chromotography using ethyl acetate-petroleum ether (2:8) as eluent. Single crystals appeared from the same eluent mixture.

### Refinement

All hydrogen atoms were placed in calculated positions with C–H = 0.93-0.98Å and refined in riding model with isotropic displacement parameters: *U*_iso_(H) = 1.5 *U*_eq_(C) for methyl group and *U*_iso_(H) = 1.2 *U*_eq_(C) for other groups.

### Crystal data

**Table:**

C_20_H_17_BrN_2_O_2_	*F*(000) = 808
*M**_r_* = 397.26	*D*_x_ = 1.553 Mg m^−^^3^
Monoclinic, *P*2_1_/*c*	Mo *K*α radiation, λ = 0.71073 Å
Hall symbol: -P 2ybc	Cell parameters from 4461 reflections
*a* = 12.5772 (4) Å	θ = 1.0–28.9°
*b* = 14.7746 (5) Å	µ = 2.44 mm^−^^1^
*c* = 9.2168 (3) Å	*T* = 295 K
β = 97.193 (2)°	Block, yellow
*V* = 1699.22 (10) Å^3^	0.30 × 0.20 × 0.20 mm
*Z* = 4	

### Data collection

**Table:**

Bruker Kappa APEXII CCD diffractometer	2831 reflections with *I* > 2σ(*I*)
Radiation source: fine-focus sealed tube	*R*_int_ = 0.041
graphite	θ_max_ = 28.9°, θ_min_ = 2.6°
ω scans	*h* = −16→17
21255 measured reflections	*k* = −19→20
4461 independent reflections	*l* = −12→12

### Refinement

**Table:**

Refinement on *F*^2^	Primary atom site location: structure-invariant direct methods
Least-squares matrix: full	Secondary atom site location: difference Fourier map
*R*[*F*^2^ > 2σ(*F*^2^)] = 0.039	Hydrogen site location: inferred from neighbouring sites
*wR*(*F*^2^) = 0.099	H-atom parameters constrained
*S* = 1.00	*w* = 1/[σ^2^(*F*_o_^2^) + (0.0406*P*)^2^ + 0.6061*P*] where *P* = (*F*_o_^2^ + 2*F*_c_^2^)/3
4461 reflections	(Δ/σ)_max_ = 0.005
227 parameters	Δρ_max_ = 0.45 e Å^−^^3^
0 restraints	Δρ_min_ = −0.38 e Å^−^^3^

### Special details

**Table:**

Geometry. All s.u.'s (except the s.u. in the dihedral angle between two l.s. planes) are estimated using the full covariance matrix. The cell s.u.'s are taken into account individually in the estimation of s.u.'s in distances, angles and torsion angles; correlations between s.u.'s in cell parameters are only used when they are defined by crystal symmetry. An approximate (isotropic) treatment of cell s.u.'s is used for estimating s.u.'s involving l.s. planes.
Refinement. Refinement of *F*^2^ against ALL reflections. The weighted *R*-factor *wR* and goodness of fit *S* are based on *F*^2^, conventional *R*-factors *R* are based on *F*, with *F* set to zero for negative *F*^2^. The threshold expression of *F*^2^ > σ(*F*^2^) is used only for calculating *R*-factors(gt) *etc*. and is not relevant to the choice of reflections for refinement. *R*-factors based on *F*^2^ are statistically about twice as large as those based on *F*, and *R*-factors based on ALL data will be even larger.

### Fractional atomic coordinates and isotropic or equivalent isotropic displacement parameters (Å^2^)

**Table:**

	*x*	*y*	*z*	*U*_iso_*/*U*_eq_	
C1	0.55355 (16)	0.06019 (14)	0.8437 (2)	0.0372 (5)	
C2	0.49922 (18)	0.02980 (16)	0.7116 (2)	0.0488 (6)	
H2	0.5295	−0.0140	0.6571	0.059*	
C3	0.4004 (2)	0.06538 (19)	0.6630 (3)	0.0590 (7)	
H3	0.3635	0.0448	0.5756	0.071*	
C4	0.35459 (19)	0.13168 (18)	0.7423 (3)	0.0577 (6)	
H4	0.2876	0.1547	0.7065	0.069*	
C5	0.40554 (19)	0.16370 (16)	0.8712 (3)	0.0501 (6)	
H5	0.3748	0.2084	0.9235	0.060*	
C6	0.50518 (17)	0.12714 (14)	0.9219 (2)	0.0403 (5)	
C7	0.5531 (2)	0.20897 (19)	1.1594 (3)	0.0710 (8)	
H7A	0.5497	0.2692	1.1201	0.107*	
H7B	0.6102	0.2053	1.2387	0.107*	
H7C	0.4864	0.1945	1.1945	0.107*	
C8	0.66164 (18)	0.09158 (15)	1.0478 (2)	0.0432 (5)	
H8	0.7191	0.0912	1.1218	0.052*	
C9	0.65405 (16)	0.03903 (13)	0.9262 (2)	0.0360 (4)	
C10	0.73020 (16)	−0.03183 (13)	0.8837 (2)	0.0361 (5)	
H10	0.6881	−0.0875	0.8665	0.043*	
C11	0.82340 (16)	−0.05514 (14)	0.9955 (2)	0.0391 (5)	
C12	0.9709 (3)	−0.12919 (19)	1.1159 (3)	0.0701 (8)	
H12A	0.9722	−0.1753	1.1911	0.084*	
H12B	1.0386	−0.1306	1.0759	0.084*	
C13	0.8786 (2)	−0.14544 (15)	0.9967 (3)	0.0513 (6)	
H13	0.8316	−0.1930	1.0265	0.062*	
C14	0.9178 (2)	−0.17035 (18)	0.8540 (3)	0.0606 (7)	
H14A	0.9755	−0.1298	0.8368	0.073*	
H14B	0.9466	−0.2313	0.8614	0.073*	
C15	0.82539 (17)	−0.07968 (15)	0.6713 (2)	0.0428 (5)	
C16	0.86662 (18)	−0.06373 (18)	0.5428 (2)	0.0532 (6)	
H16	0.8988	−0.1106	0.4971	0.064*	
C17	0.86065 (18)	0.0213 (2)	0.4810 (2)	0.0547 (6)	
H17	0.8874	0.0322	0.3930	0.066*	
C18	0.81440 (17)	0.08935 (17)	0.5523 (2)	0.0456 (5)	
C19	0.77092 (16)	0.07430 (14)	0.6800 (2)	0.0375 (5)	
H19	0.7387	0.1215	0.7251	0.045*	
C20	0.77544 (15)	−0.01140 (14)	0.7409 (2)	0.0351 (4)	
N1	0.57283 (15)	0.14517 (12)	1.0459 (2)	0.0453 (4)	
N2	0.86495 (15)	−0.00096 (15)	1.0909 (2)	0.0554 (5)	
O1	0.95362 (15)	−0.04308 (15)	1.17437 (19)	0.0769 (6)	
O2	0.83522 (14)	−0.16586 (11)	0.73157 (18)	0.0571 (4)	
Br1	0.80960 (3)	0.20837 (2)	0.47468 (3)	0.07806 (14)	

### Atomic displacement parameters (Å^2^)

**Table:**

	*U*^11^	*U*^22^	*U*^33^	*U*^12^	*U*^13^	*U*^23^
C1	0.0354 (11)	0.0388 (11)	0.0401 (11)	−0.0033 (9)	0.0158 (9)	0.0016 (9)
C2	0.0451 (13)	0.0560 (14)	0.0472 (12)	−0.0074 (11)	0.0133 (11)	−0.0055 (10)
C3	0.0435 (14)	0.0775 (18)	0.0559 (14)	−0.0078 (13)	0.0055 (11)	−0.0018 (13)
C4	0.0366 (13)	0.0696 (17)	0.0678 (16)	0.0011 (12)	0.0101 (12)	0.0145 (14)
C5	0.0419 (13)	0.0494 (13)	0.0634 (15)	0.0022 (11)	0.0233 (12)	0.0084 (11)
C6	0.0380 (12)	0.0398 (11)	0.0460 (12)	−0.0036 (9)	0.0167 (10)	0.0026 (9)
C7	0.0712 (19)	0.0711 (18)	0.0731 (18)	0.0136 (14)	0.0177 (15)	−0.0307 (14)
C8	0.0414 (12)	0.0468 (12)	0.0428 (11)	0.0006 (10)	0.0108 (9)	−0.0070 (9)
C9	0.0380 (11)	0.0338 (10)	0.0386 (10)	−0.0034 (9)	0.0147 (9)	−0.0006 (8)
C10	0.0404 (11)	0.0326 (10)	0.0373 (10)	−0.0035 (9)	0.0123 (9)	−0.0023 (8)
C11	0.0416 (12)	0.0413 (12)	0.0369 (11)	−0.0009 (9)	0.0155 (9)	0.0031 (9)
C12	0.080 (2)	0.0640 (18)	0.0634 (16)	0.0128 (15)	−0.0033 (15)	0.0193 (14)
C13	0.0583 (15)	0.0387 (12)	0.0571 (14)	0.0024 (11)	0.0079 (12)	0.0104 (10)
C14	0.0653 (17)	0.0479 (14)	0.0683 (16)	0.0204 (13)	0.0070 (14)	−0.0012 (12)
C15	0.0377 (12)	0.0474 (13)	0.0429 (11)	0.0012 (10)	0.0038 (9)	−0.0121 (10)
C16	0.0411 (13)	0.0760 (18)	0.0442 (12)	0.0020 (12)	0.0116 (10)	−0.0231 (12)
C17	0.0385 (13)	0.094 (2)	0.0334 (11)	−0.0071 (13)	0.0114 (9)	−0.0054 (12)
C18	0.0342 (12)	0.0657 (15)	0.0368 (11)	−0.0094 (10)	0.0048 (9)	0.0049 (10)
C19	0.0333 (11)	0.0456 (12)	0.0341 (10)	−0.0025 (9)	0.0065 (8)	−0.0032 (9)
C20	0.0304 (10)	0.0428 (11)	0.0325 (10)	−0.0022 (9)	0.0057 (8)	−0.0069 (8)
N1	0.0453 (11)	0.0444 (10)	0.0488 (10)	0.0039 (8)	0.0158 (9)	−0.0110 (8)
N2	0.0421 (11)	0.0735 (14)	0.0504 (11)	0.0127 (10)	0.0054 (9)	−0.0159 (10)
O1	0.0582 (11)	0.1117 (16)	0.0574 (10)	0.0313 (11)	−0.0067 (9)	−0.0287 (11)
O2	0.0644 (11)	0.0422 (9)	0.0642 (10)	0.0073 (8)	0.0056 (9)	−0.0151 (8)
Br1	0.0801 (2)	0.0842 (2)	0.0702 (2)	−0.01674 (16)	0.01064 (15)	0.03257 (15)

### Geometric parameters (Å, °)

**Table:**

C1—C2	1.394 (3)	C11—N2	1.254 (3)
C1—C6	1.407 (3)	C11—C13	1.503 (3)
C1—C9	1.426 (3)	C12—O1	1.409 (3)
C2—C3	1.371 (3)	C12—C13	1.514 (4)
C2—H2	0.9300	C12—H12A	0.9700
C3—C4	1.390 (4)	C12—H12B	0.9700
C3—H3	0.9300	C13—C14	1.507 (3)
C4—C5	1.362 (4)	C13—H13	0.9800
C4—H4	0.9300	C14—O2	1.436 (3)
C5—C6	1.391 (3)	C14—H14A	0.9700
C5—H5	0.9300	C14—H14B	0.9700
C6—N1	1.363 (3)	C15—C16	1.371 (3)
C7—N1	1.453 (3)	C15—C20	1.388 (3)
C7—H7A	0.9600	C15—O2	1.389 (3)
C7—H7B	0.9600	C16—C17	1.378 (4)
C7—H7C	0.9600	C16—H16	0.9300
C8—C9	1.356 (3)	C17—C18	1.370 (3)
C8—N1	1.367 (3)	C17—H17	0.9300
C8—H8	0.9300	C18—C19	1.377 (3)
C9—C10	1.504 (3)	C18—Br1	1.897 (2)
C10—C11	1.501 (3)	C19—C20	1.383 (3)
C10—C20	1.528 (3)	C19—H19	0.9300
C10—H10	0.9800	N2—O1	1.417 (3)
			
C2—C1—C6	118.6 (2)	C13—C12—H12A	110.5
C2—C1—C9	134.5 (2)	O1—C12—H12B	110.5
C6—C1—C9	106.97 (18)	C13—C12—H12B	110.5
C3—C2—C1	119.1 (2)	H12A—C12—H12B	108.7
C3—C2—H2	120.4	C11—C13—C14	114.31 (19)
C1—C2—H2	120.4	C11—C13—C12	100.1 (2)
C2—C3—C4	121.1 (2)	C14—C13—C12	111.6 (2)
C2—C3—H3	119.4	C11—C13—H13	110.2
C4—C3—H3	119.4	C14—C13—H13	110.2
C5—C4—C3	121.5 (2)	C12—C13—H13	110.2
C5—C4—H4	119.2	O2—C14—C13	113.0 (2)
C3—C4—H4	119.2	O2—C14—H14A	109.0
C4—C5—C6	117.6 (2)	C13—C14—H14A	109.0
C4—C5—H5	121.2	O2—C14—H14B	109.0
C6—C5—H5	121.2	C13—C14—H14B	109.0
N1—C6—C5	130.4 (2)	H14A—C14—H14B	107.8
N1—C6—C1	107.61 (18)	C16—C15—C20	121.1 (2)
C5—C6—C1	122.0 (2)	C16—C15—O2	118.6 (2)
N1—C7—H7A	109.5	C20—C15—O2	120.29 (19)
N1—C7—H7B	109.5	C15—C16—C17	120.5 (2)
H7A—C7—H7B	109.5	C15—C16—H16	119.8
N1—C7—H7C	109.5	C17—C16—H16	119.8
H7A—C7—H7C	109.5	C18—C17—C16	118.4 (2)
H7B—C7—H7C	109.5	C18—C17—H17	120.8
C9—C8—N1	110.37 (19)	C16—C17—H17	120.8
C9—C8—H8	124.8	C17—C18—C19	122.0 (2)
N1—C8—H8	124.8	C17—C18—Br1	119.66 (16)
C8—C9—C1	106.33 (18)	C19—C18—Br1	118.37 (18)
C8—C9—C10	129.23 (19)	C18—C19—C20	119.6 (2)
C1—C9—C10	124.37 (18)	C18—C19—H19	120.2
C11—C10—C9	116.69 (17)	C20—C19—H19	120.2
C11—C10—C20	107.08 (16)	C19—C20—C15	118.41 (18)
C9—C10—C20	113.88 (16)	C19—C20—C10	121.97 (17)
C11—C10—H10	106.1	C15—C20—C10	119.59 (18)
C9—C10—H10	106.1	C6—N1—C8	108.71 (17)
C20—C10—H10	106.1	C6—N1—C7	125.9 (2)
N2—C11—C10	123.8 (2)	C8—N1—C7	125.4 (2)
N2—C11—C13	114.2 (2)	C11—N2—O1	109.3 (2)
C10—C11—C13	121.93 (19)	C12—O1—N2	109.74 (18)
O1—C12—C13	106.2 (2)	C15—O2—C14	112.10 (18)
O1—C12—H12A	110.5		
			
C6—C1—C2—C3	−0.6 (3)	C12—C13—C14—O2	−166.5 (2)
C9—C1—C2—C3	179.6 (2)	C20—C15—C16—C17	1.0 (3)
C1—C2—C3—C4	0.8 (4)	O2—C15—C16—C17	−178.6 (2)
C2—C3—C4—C5	−0.2 (4)	C15—C16—C17—C18	1.1 (3)
C3—C4—C5—C6	−0.5 (3)	C16—C17—C18—C19	−2.3 (3)
C4—C5—C6—N1	−179.3 (2)	C16—C17—C18—Br1	177.58 (17)
C4—C5—C6—C1	0.7 (3)	C17—C18—C19—C20	1.3 (3)
C2—C1—C6—N1	179.84 (18)	Br1—C18—C19—C20	−178.55 (15)
C9—C1—C6—N1	−0.3 (2)	C18—C19—C20—C15	0.8 (3)
C2—C1—C6—C5	−0.2 (3)	C18—C19—C20—C10	178.98 (18)
C9—C1—C6—C5	179.70 (19)	C16—C15—C20—C19	−1.9 (3)
N1—C8—C9—C1	−0.7 (2)	O2—C15—C20—C19	177.61 (18)
N1—C8—C9—C10	−177.85 (19)	C16—C15—C20—C10	179.84 (19)
C2—C1—C9—C8	−179.5 (2)	O2—C15—C20—C10	−0.6 (3)
C6—C1—C9—C8	0.6 (2)	C11—C10—C20—C19	−113.4 (2)
C2—C1—C9—C10	−2.2 (4)	C9—C10—C20—C19	17.1 (3)
C6—C1—C9—C10	177.92 (17)	C11—C10—C20—C15	64.8 (2)
C8—C9—C10—C11	6.3 (3)	C9—C10—C20—C15	−164.68 (18)
C1—C9—C10—C11	−170.40 (18)	C5—C6—N1—C8	179.9 (2)
C8—C9—C10—C20	−119.3 (2)	C1—C6—N1—C8	−0.1 (2)
C1—C9—C10—C20	64.0 (2)	C5—C6—N1—C7	1.3 (4)
C9—C10—C11—N2	−28.3 (3)	C1—C6—N1—C7	−178.7 (2)
C20—C10—C11—N2	100.7 (2)	C9—C8—N1—C6	0.5 (2)
C9—C10—C11—C13	154.99 (18)	C9—C8—N1—C7	179.1 (2)
C20—C10—C11—C13	−76.1 (2)	C10—C11—N2—O1	−177.52 (18)
N2—C11—C13—C14	−122.5 (2)	C13—C11—N2—O1	−0.6 (3)
C10—C11—C13—C14	54.6 (3)	C13—C12—O1—N2	−6.2 (3)
N2—C11—C13—C12	−3.1 (3)	C11—N2—O1—C12	4.4 (3)
C10—C11—C13—C12	173.93 (19)	C16—C15—O2—C14	103.6 (2)
O1—C12—C13—C11	5.4 (3)	C20—C15—O2—C14	−75.9 (3)
O1—C12—C13—C14	126.8 (2)	C13—C14—O2—C15	87.0 (3)
C11—C13—C14—O2	−53.8 (3)		

### Hydrogen-bond geometry (Å, °)

**Table:**

Cg1and Cg2 are the centroids of the C1–C6 and C15–C20 rings, respectively.

**Table:**

*D*—H···*A*	*D*—H	H···*A*	*D*···*A*	*D*—H···*A*
C17—H17···O1^i^	0.93	2.53	3.329 (3)	144
C3—H3···Cg2^ii^	0.93	2.79	3.653 (3)	155
C7—H7A···Cg1^iii^	0.96	2.92	3.427 (3)	114

Symmetry codes: (i) *x*, *y*, *z*−1; (ii) −*x*+1, −*y*, −*z*+1; (iii) *x*, −*y*−1/2, *z*−1/2.
